# ERAS-based sequential olive oil-lactulose protocol in day-case laparoscopic inguinal hernia repair: a prospective comparison

**DOI:** 10.3389/fsurg.2026.1733411

**Published:** 2026-02-18

**Authors:** Xiaoqin Zhou, Penghui Liu, Gengyu Tong Zhao, Deyan Bai, Wenqing Liu, Yueqian Zeng, Run Wan, Huijia Pan, Jie Mao

**Affiliations:** 1 The General Surgery Department, Lanzhou University Second Hospital, Lanzhou, China; 2 Northeastern University, Boston, MA, United States

**Keywords:** bowel preparation, day-case surgery, enhanced recovery after surgery (ERAS), inguinal hernia, lactulose, olive oil

## Abstract

**Objective:**

To investigate the effect of a sequential olive oil–lactulose approach within an enhanced recovery after surgery (ERAS) pathway on perioperative bowel management and postoperative recovery in patients undergoing day-case laparoscopic inguinal hernia repair.

**Methods:**

A total of 204 patients who underwent day-case laparoscopic inguinal hernia repair between June 2024 and June 2025 were divided into two groups according to bowel-preparation regimen: a study group (*n* = 102) and a control group (*n* = 102). The study group received a sequential regimen of olive oil and lactulose bowel preparation. The control group underwent traditional polyethylene glycol (PEG) bowel preparation. Both groups received standard ERAS measures. Outcomes compared between groups included bowel-preparation compliance and tolerance, gastrointestinal recovery (time to first flatus and defecation), time to first ambulation, length of hospital stay, incidence of complications, 24-h postoperative pain score by visual analogue scale (VAS), and discharge satisfaction.

**Results:**

The study group showed a significantly higher bowel-preparation compliance and tolerance than the control group (100% vs. 95.1%; 96.1% vs. 68.6%; *P* < 0.05). Compared with the control group, the study group had shorter times to first flatus (14.2 ± 3.1 h vs. 22.7 ± 5.3 h), first defecation (18.4 ± 4.2 h vs. 27.1 ± 6.3 h), first ambulation (8.9 ± 2.3 h vs. 14.1 ± 3.4 h), and length of hospital stay (1.8 ± 0.6 d vs. 2.7 ± 0.9 d) (all *P* < 0.001). The incidences of abdominal distension (10.8% vs. 70.6%), nausea/vomiting (7.8% vs. 35.3%), and dry mouth (21.6% vs. 44.1%) were lower in the study group (both *P* < 0.001), whereas the rate of urinary retention did not differ significantly between groups (*P* > 0.05). Postoperatively, the study group had lower VAS pain scores (2.1 ± 0.7 vs. 3.8 ± 1.1) and higher satisfaction scores (4.3 ± 0.7 vs. 2.9 ± 0.8) (both *P* < 0.001).

**Conclusions:**

Within the ERAS pathway, the sequential approach of olive oil and lactulose significantly improved bowel preparation compliance and tolerance in patients undergoing daytime laparoscopic inguinal hernia repair. This approach accelerated gastrointestinal function recovery, shortened hospital stays, reduced complication rates, alleviated postoperative pain, and enhanced patient satisfaction.

## 1 Introduction

Inguinal hernia is one of the most common conditions in general surgery, with an annual incidence of approximately 1.5‰, and day-case procedures now account for more than 65% of all cases (1). In such operations, traditional bowel preparation is usually performed with polyethylene glycol (PEG) electrolyte solution. This regimen requires patients to ingest 2,000–3,000 mL of fluid within a short preoperative period, which is often poorly tolerated and frequently accompanied by adverse reactions such as nausea, vomiting, and abdominal distension (2, 3), thereby hindering the achievement of enhanced recovery after surgery (ERAS) goals.

The concept of ERAS emphasizes multidisciplinary collaboration to optimize perioperative management, with preoperative nutritional support and bowel function regulation being key components (4). Recent studies have shown that lactulose, as an osmotic laxative, can be fermented by gut microbiota in the colon to produce short-chain fatty acid (SCFA), thereby stimulating intestinal peristalsis and improving bowel motility (5). Olive oil, which is rich in monounsaturated fatty acids such as oleic acid, can trigger the gastrocolic reflex, promoting gastric emptying and defecation (6). Previous evidence suggests that both agents demonstrate certain advantages in preoperative bowel preparation, but their application remains limited by incomplete efficacy or insufficient coverage.

Based on the above evidence, this study innovatively proposed and evaluated a sequential nutritional protocol consisting of preoperative olive oil priming, preoperative lactulose reinforcement, and postoperative olive oil maintenance, embedded within the ERAS pathway. This protocol was designed to enhance bowel-preparation tolerance, reduce adverse effects, and accelerate postoperative gastrointestinal recovery in patients undergoing day-case laparoscopic inguinal hernia repair through a continuous “priming–purgation–restoration” mechanism, thereby providing new evidence for optimizing perioperative bowel management.

Mechanistically, triglycerides in olive oil are hydrolyzed to 2-monoacylglycerols (notably 2-oleoyl glycerol, 2-OG) and free fatty acids. 2-OG can activate GPR119 on enteroendocrine L cells and stimulate incretin secretion (including GLP-1) in humans (7). However, GLP-1 receptor signaling is generally associated with delayed gastric emptying and slower gastrointestinal transit; therefore, any bowel-facilitating effect of olive oil in our setting is more plausibly related to postprandial neurohormonal responses (e.g., gastrocolonic response) and improved regimen tolerability rather than a direct GLP-1–driven pro-motility mechanism (8, 9). Lactulose, a non-absorbable disaccharide, exerts an osmotic effect and acts as a prebiotic substrate for colonic bacteria, increasing SCFA production, which may support epithelial barrier integrity and modulate postoperative inflammation (10).

## 2 Material & methods

### 2.1 Study population

A total of 204 patients who underwent day-case laparoscopic inguinal hernia repair at the Second Hospital of Lanzhou University between June 2024 and June 2025 were enrolled in this study. Patients were assigned to either the study group (*n* = 102) or the control group (*n* = 102) according to the bowel-preparation protocol. Inclusion criteria: (1) Age ≥ 18 years; (2) Diagnosis of primary unilateral inguinal hernia (Gilbert type I-III); (3) American Society of Anesthesiologists (ASA) physical status classification I-II; (4) Body mass index (BMI) 18.5–35.0 kg/m²; (5) Scheduled for day-case laparoscopic inguinal hernia repair; (6) Clear consciousness and normal communication ability. Exclusion criteria: (1) Lactase deficiency or fructose intolerance; (2) Severe cardiac, hepatic, or renal dysfunction; (3) Previous history of abdominal surgery; (4) Cognitive impairment, unable to cooperate.

The study protocol was approved by the Ethics Committee of Lanzhou University Second Hospital (Approval No. 2024A-1454), and written informed consent was obtained from all participants prior to enrollment.

### 2.2 Methods

Standardized preoperative low-residue/low-fiber diet instruction (both groups). As part of the institutional ERAS pathway, all participants received the same standardized dietary instruction for the 3 d before surgery: a low-residue (low-fiber) diet was recommended to minimize stool bulk. Patients were advised to avoid high-fiber foods, including whole grains/coarse cereals, legumes, nuts and seeds, and most raw vegetables and fruits (especially those with skins/seeds). Patients were encouraged to consume low-fiber options such as lean meat/fish, eggs, tofu, and clear liquids, with adequate hydration encouraged. Habitual dietary pattern (e.g., predominantly vegetarian intake) was not formally recorded, and baseline fecal loading/constipation status was not routinely assessed by plain abdominal radiography or used for stratification (Table 1).

**Table 1:** Bowel-preparation protocols of the two groups.

Study phase	Control group	Study group	Common ERAS measures
3 d before surgery	Low-residue (low-fiber) diet (avoid high-fiber foods; maintain hydration)	Low-residue (low-fiber) diet + Olive oil 30 mL orally, tid	(1) Multidisciplinary health education (surgeons, nurses, pharmacists, dietitians) (2) Pain management planning (3) Nutritional risk screening (NRS 2002)
1 d before surgery	PEG 68.56 g × 2 sachets + 3,000 mL warm water (divided doses)	Lactulose 30 mL orally, tid + 500 mL warm water	(1) Fasting for 6 h and fluid restriction for 2 h preoperatively (2) Psychological support
d of surgery	Routine postoperative care	Olive oil 30 mL orally, tid starting ∼6 h after anesthesia recovery	(1) Multimodal analgesia (NSAIDs + local ice packs) (2) Head-of-bed elevation to 45° after recovery
Postoperative d 1–3	Standard discharge guidance	Olive oil 30 mL orally, tid	(1) Early mobilization (2) Monitoring and management of complications (3) Structured discharge education

h, hour; d, day; ERAS, enhanced recovery after surgery; tid, three times daily; PEG, polyethylene glycol; NSAIDs, nonsteroidal anti-inflammatory drugs. Postoperative olive oil maintenance was an ERAS adjunct and was not included in the bowel-preparation compliance endpoint, which was defined based on completion of the preoperative preparation components.

Control group. Patients received conventional polyethylene glycol (PEG) bowel preparation combined with routine care. 1 d before surgery, PEG electrolyte powder (68.56 g/sachet; 2 sachets) was dissolved in 3,000 mL of warm water and taken in divided doses to complete bowel cleansing. On the day of surgery and 1–2 d postoperatively, patients received routine postoperative care and rehabilitation guidance.

Study group. Patients received a sequential olive oil–lactulose regimen within the ERAS pathway. During the 3 d before surgery, patients followed the standardized low-residue (low-fiber) diet described above and additionally received olive oil (acidity ≤ 0.8%) 30 mL orally, three times daily. 1 d before surgery, lactulose solution 30 mL (diluted in 100 mL warm water) was administered orally three times daily, accompanied by an additional 500 mL warm water, and patients were instructed to complete all doses as scheduled. Approximately 6 h after recovery from anesthesia, olive oil 30 mL orally three times daily was resumed. On postoperative d 1–2 (or until discharge), olive oil 30 mL three times daily was continued.

ERAS perioperative measures. Both groups received standardized ERAS management implemented by a multidisciplinary team (surgeons, anesthesiologists, specialized nurses, dietitians, and pharmacists). Key measures included preoperative health education and Nutritional Risk Screening 2002 (NRS 2002), multimodal analgesia, head-of-bed elevation and early mobilization after surgery, monitoring and management of complications, and structured discharge education.

### 2.3 Outcomes

#### 2.3.1 Compliance and tolerance of bowel preparation

Compliance: defined as whether the patient completed the prespecified preoperative bowel-preparation components. Specifically, compliance required completion of: (i) the full PEG regimen on postoperative d −1 in the control group; or (ii) olive oil administration (tid) during the 3 d before surgery and lactulose administration (tid) on postoperative d −1 in the study group. Postoperative continuation of olive oil was considered an ERAS adjunct and was not included in the compliance endpoint. In this day-case setting, the preoperative regimen was performed largely at home; therefore, compliance was assessed by structured patient self-report at admission.

Tolerance: defined as absence of vomiting during the preparation process.

#### 2.3.2 Gastrointestinal recovery

Time to first flatus and first defecation after surgery (h).

#### 2.3.3 Postoperative recovery indicators

Time to first ambulation (h) and length of hospital stay (d).

#### 2.3.4 Complications

Incidence of postoperative abdominal distension, nausea/vomiting, dry mouth, and urinary retention. Colicky abdominal pain/cramping during preparation was not predefined as a complication endpoint and was therefore not quantitatively analyzed in the present study.

#### 2.3.5 Pain assessment

Postoperative pain was assessed using the visual analogue scale (VAS, 0–10, higher scores indicating greater pain intensity) at 6 h, 24 h, and 48 h after surgery. This study mainly reports the 24-h VAS score.

#### 2.3.6 Patient satisfaction

Overall satisfaction with perioperative management was assessed at discharge using a 5-point Likert scale (1 = very dissatisfied, 5 = very satisfied).

### 2.4 Statistical analysis

Statistical analysis were performed using SPSS version 26.0 (IBM Corp., Armonk, NY, USA). Continuous variables with normal distribution were expressed as mean ± standard deviation (mean ± SD) and compared between groups using the independent-samples *t*-test (or *t*-test in the case of unequal variances). Categorical variables were presented as frequencies and percentages, and group comparisons were performed using the *χ*² test or Fisher's exact test. A two-sided *P* value < 0.05 was considered statistically significant.

## 3 Results

### 3.1 Baseline characteristics

There were no significant differences between the two groups in terms of age, sex, BMI, or hernia classification (*P* > 0.05), indicating good comparability (Table 2).

**Table 2:** Baseline characteristics of patients in the two groups.

Variables	Control group (*n* = 102)	Study group (*n* = 102)	*P*
Age (mean ± SD)	68.3 ± 7.1	69.2 ± 6.9	0.387
BMI (mean ± SD)	23.8 ± 3.4	24.1 ± 3.2	0.472
Gender [*n* (%)]			0.674
Male	87 (85.3)	89 (87.3)	
Female	15 (14.7)	13 (12.7)	
Gilbert classification [n (%)]			0.796
Type I	32 (31.4)	35 (34.3)	
Type II	45 (44.1)	42 (41.2)	
Type III	25 (24.5)	25 (24.5)	

Age, years; BMI, body mass index (kg/m²); *n*, number; SD, standard deviation.

### 3.2 Bowel-preparation compliance, tolerance, and complications between the two groups

Patients in the study group had significantly higher bowel-preparation compliance and tolerance than those in the control group (*P* < 0.05). The incidences of abdominal distension, nausea/vomiting, and dry mouth were significantly lower in the study group than in the control group (*P* < 0.001). No significant difference was observed in the incidence of urinary retention (*P* > 0.05) (Table 3).

**Table 3:** Bowel-preparation compliance, tolerance, and complications between the two groups.

Variables	Control group (*n* = 102)	Study group (*n* = 102)	*P*
Compliance [*n* (%)]	97 (95.1%)	102 (100%)	0.043
Tolerance [*n* (%)]	70 (68.6%)	98 (96.1%)	<0.001
Abdominal distension [*n* (%)]	72 (70.6%)	11 (10.8%)	<0.001
Nausea/vomiting [*n* (%)]	36 (35.3%)	8 (7.8%)	<0.001
Dry mouth [*n* (%)]	45 (44.1%)	22 (21.6%)	<0.001
Urinary retention [*n* (%)]	8 (7.8%)	5 (4.9%)	0.382

*n*, number.

### 3.3 Gastrointestinal and clinical recovery between the two groups

Patients in the study group experienced significantly earlier postoperative gastrointestinal recovery, as indicated by shorter times to first flatus, first defecation, and first ambulation, compared with the control group. In addition, the length of hospital stay was significantly reduced in the study group. All differences were statistically significant (*P* < 0.001) (Table 4).

**Table 4:** Gastrointestinal and clinical recovery between the two groups.

Variables	Control group (*n* = 102)	Study group (*n* = 102)	*P*
First Flatus (h)	22.7 ± 5.3	14.2 ± 3.1	<0.001
First Defecation (h)	27.1 ± 6.3	18.4 ± 4.2	<0.001
First Ambulation (h)	14.1 ± 3.4	8.9 ± 2.3	<0.001
Length of hospital stay (d)	2.7 ± 0.9	1.8 ± 0.6	<0.001

h, hour; d, day; *n*, number.

### 3.4 Postoperative pain and patient satisfaction between the two groups

The study group had significantly lower VAS pain scores at 24 h postoperatively and higher satisfaction scores at discharge compared with the control group (*P* < 0.001) (Table 5).

**Table 5:** Postoperative pain and satisfaction between the two groups.

Variables	Control group (*n* = 102)	Study group (*n* = 102)	*P*
VAS pain score at 24 h (mean ± SD)	3.8 ± 1.1	2.1 ± 0.7	<0.001
Satisfaction score at discharge (mean ± SD)	2.9 ± 0.8	4.3 ± 0.7	<0.001

h, hour; *n*, number; SD, standard deviation.

## 4 Discussion

### 4.1 Improvement in compliance and tolerance

This study demonstrated that patients in the study group exhibited significantly higher bowel-preparation compliance and tolerance compared with the control group (compliance: 100% vs. 95.1%; tolerance: 96.1% vs. 68.6%, *P* < 0.05). These findings suggest that the sequential olive oil–lactulose protocol offers clear advantages in terms of patient acceptance and tolerability. Conventional PEG regimens require ingestion of approximately 3,000 mL of solution within a short preoperative period, which often induces nausea, vomiting, and abdominal distension, leading to poor compliance—particularly among elderly patients (3). Previous studies have confirmed that although high-volume PEG provides reliable cleansing, it is associated with poor patient willingness for repeat use and generally low compliance (11).

In recent years, modified regimens have gained increasing attention. Some studies have shown that preoperative administration of olive oil in combination with PEG significantly improves colonic cleansing quality and enhances patient satisfaction (12), while others have reported that olive oil plus low-dose PEG achieves better compliance and tolerance than conventional PEG alone (13). Lactulose, as an osmotic laxative, has also been validated in several studies to improve the overall patient experience during bowel preparation (14, 15). In contrast to these earlier approaches, our study employed a sequential combination of olive oil and lactulose embedded within an ERAS pathway, achieving dual improvements in compliance and tolerance. Olive oil, with its relatively acceptable taste and administration in small, divided doses, may improve patient acceptance and reduce discomfort related to rapid large-volume fluid intake. In addition, dietary fat can trigger postprandial neurohormonal responses (the gastrocolonic response), which may support colonic motility and facilitate bowel emptying (16), while lactulose is fermented by the gut microbiota to generate short-chain fatty acids, gently promoting peristalsis and avoiding the acute gastrointestinal irritation associated with high-volume PEG (17). Moreover, the administration in small, divided doses further reduced gastrointestinal burden. In addition, comprehensive health education and preoperative psychological support within the ERAS framework enhanced patient awareness and participation, facilitating adherence to the regimen. Overall, the sequential olive oil–lactulose protocol not only demonstrated superior compliance and tolerance in clinical outcomes but also improved patient experience, aligning well with the ERAS principles of “fast, safe, and comfortable” recovery, particularly benefiting elderly and otherwise vulnerable populations. Notably, the two regimens differ in duration and supervision intensity. PEG is typically administered over a single preoperative day and, in some settings, may be more readily supervised, whereas the sequential protocol spans multiple perioperative days. To improve comparability, we defined “compliance” primarily as completion of the preoperative bowel-preparation components. Nevertheless, because adherence was based on self-report in a day-case context, differential adherence bias cannot be fully excluded. Future randomized trials should incorporate standardized adherence monitoring (e.g., supervised dosing when feasible, electronic diaries, or verification of returned sachets/bottles) to strengthen the validity of compliance comparisons.

### 4.2 Reduction of complications and improvement of safety

This study found that the incidences of postoperative abdominal distension, nausea/vomiting, and dry mouth were significantly lower in the study group than in the control group. These benefits may be attributed to the dual physiological and experiential advantages of the sequential olive oil–lactulose protocol. Conventional PEG solution, characterized by its bitter taste and requirement for large-volume ingestion within a short time, often provokes nausea or vomiting in some patients, thereby compromising compliance and tolerance. In contrast, improving the taste can significantly reduce these adverse reactions.

From a mechanistic perspective, extra virgin olive oil (EVOO) has been shown to exert anti-inflammatory effects, notably downregulating pro-inflammatory cytokines such as Interleukin-1β and Interleukin-6, thereby alleviating intestinal inflammation and reducing bloating-related symptoms (18). Unlike potent purgatives, lactulose acts primarily via osmotic mechanisms to lower intraluminal pH and is fermented by gut microbiota into short-chain fatty acids, which gently stimulate peristalsis. This process helps minimize disruption to the intestinal barrier and contributes to lower rates of abdominal distension (19). It should be noted that lactulose reaches the colon largely unchanged and is metabolized by colonic bacteria into organic acids (including short-chain fatty acids) and gases; therefore, both its laxative efficacy and tolerability may vary with baseline microbial conditions (20). Abdominal cramping/colicky pain, bloating, and flatulence are recognized adverse effects related to fermentation. Moreover, in patients with suspected small intestinal bacterial overgrowth (SIBO) or altered transit, lactulose may be fermented more proximally, which could theoretically affect predictability of response and symptom burden (21). Consequently, the sequential regimen avoids the acute microbial and mucosal disturbances associated with high-volume, rapid PEG-induced catharsis, thereby reducing the risk of nausea, vomiting, and bloating at their root.

Furthermore, the dry mouth observed in PEG regimens does not reflect true dehydration but rather results from repeated ingestion of large volumes of unpleasant-tasting fluid, leading to “taste fatigue” and psychological aversion. The olive oil–lactulose protocol, by markedly reducing fluid intake and offering more acceptable taste characteristics, significantly lowered the incidence of reported dry mouth. The incidence of urinary retention did not differ significantly between groups, which was expected. Postoperative urinary retention is a common complication after inguinal hernia repair, usually related to surgical dissection and nerve traction, local edema, type of anesthesia (particularly spinal anesthesia), postoperative pain, or pre-existing conditions such as prostatic hyperplasia, rather than the method of bowel preparation itself.

### 4.3 Promotion of gastrointestinal function and clinical recovery

In this study, patients in the study group demonstrated significant advantages over the control group in terms of time to first flatus, time to first defecation, time to first ambulation, and length of hospital stay (all *P* < 0.001). These improvements may be attributed to the integrated “priming–purgation–restoration” framework underlying the sequential olive oil–lactulose protocol.

First, regarding “priming,” it should be acknowledged that dietary triglycerides are predominantly emulsified, hydrolyzed, and absorbed in the upper small intestine; therefore, a consistent direct colonic luminal “lubrication” effect from orally administered olive oil is uncertain and may vary across individuals (22). A more plausible explanation is that lipid exposure triggers postprandial neurohormonal responses that enhance colonic motor activity (i.e., the gastrocolonic response), in which cholecystokinin-mediated and neural pathways have been implicated (23). In addition, olive oil digestion generates 2-monoacylglycerols (notably 2-oleoyl glycerol, 2-OG) and free fatty acids; 2-OG is a GPR119 agonist on enteroendocrine L cells and can stimulate GLP-1 release in humans (24). However, because GLP-1 signaling is generally associated with delayed gastric emptying and reduced gastrointestinal transit, its role in accelerating early bowel function recovery is likely indirect and reflects broader postprandial coordination rather than a simple pro-motility mechanism (25).

Second, for “purgation,” lactulose acts as an osmotic prebiotic and is fermented by gut microbiota into short-chain fatty acids, which lower intraluminal pH and gently stimulate peristalsis, thereby facilitating bowel emptying (19). Dynamic MRI evidence further supports that lactulose increases small bowel water content and enhances intestinal motility, indicating that its laxative effect involves both osmotic and physiological stimulation (26). Beyond its osmotic effect, lactulose may contribute to postoperative recovery through microbiota–SCFA–barrier/immune pathways, including improving epithelial energy supply (especially via butyrate), enhancing tight-junction integrity and mucus production, and attenuating gut-derived inflammatory signaling after surgery (27, 28).

Third, for “restoration,” postoperative olive oil intake may provide mucosa-protective and anti-inflammatory effects through its minor phenolic compounds and monounsaturated fatty acids, potentially creating a more favorable milieu for early recovery of bowel function (29).

Finally, we acknowledge the reviewer's point that PEG-based solutions remain highly reliable for bowel cleansing because PEG is non-absorbable and provides an iso-osmotic washout effect within the intestinal lumen (30). Nevertheless, the large required volume and poor palatability may contribute to perioperative discomfort in some patients, which could partly explain the inferior tolerance and recovery indicators observed in the control group.

### 4.4 Reduction of postoperative pain and improvement of patient satisfaction

Patients in the study group had significantly lower VAS pain scores at 24 h postoperatively and higher satisfaction scores at discharge compared with the control group. This improvement can be attributed not only to the standardized multimodal analgesia embedded in the ERAS pathway but also to the beneficial effects of the sequential olive oil–lactulose protocol on postoperative bowel function. Abdominal distension and nausea are common discomforts following inguinal hernia surgery, which can significantly exacerbate the perception of incisional pain. Lactulose may help mitigate distension-related visceral discomfort by facilitating bowel transit and increasing SCFA production, which in turn may reduce intraluminal pressure and improve postoperative gut comfort. Beyond motility, SCFAs—particularly butyrate—serve as an energy source for colonocytes and are associated with enhanced tight-junction integrity and mucin production, thereby strengthening the mucosal barrier and potentially reducing endotoxin-driven inflammation after surgery. SCFAs can also signal through SCFA-sensing receptors to modulate immune responses, which provides a plausible biological link between microbiota-mediated fermentation and improved postoperative comfort (27, 28).

Previous studies have confirmed that postoperative gastrointestinal dysmotility exacerbates pain perception and adverse emotional responses, whereas improved bowel function can significantly reduce pain intensity (31). Moreover, polyphenolic compounds (e.g., hydroxytyrosol) and monounsaturated fatty acids in olive oil exert anti-inflammatory effects by downregulating the cyclooxygenase-2/prostaglandin E2 pathway and inhibiting pro-inflammatory cytokines such as Tumor Necrosis Factor-α and Interleukin-6 (29). Lactulose, by modulating gut microbiota composition and enhancing mucosal barrier integrity, indirectly reduces the translocation of inflammatory mediators into the systemic circulation, thereby further attenuating pain-related inflammatory responses (27, 28). Collectively, these synergistic effects explain the superior pain control and higher patient satisfaction observed in the study group, underscoring the importance of combining nutritional modulation with ERAS-based multimodal analgesia to optimize recovery in day-case surgery.

The sequential olive oil–lactulose protocol demonstrated significant advantages over the conventional PEG regimen across multiple dimensions, including compliance, tolerance, complication control, gastrointestinal recovery, pain relief, and patient satisfaction. Unlike previous approaches that applied single or combined agents only preoperatively, this study innovatively extended the use of olive oil and lactulose throughout the entire perioperative period, establishing a closed-loop management mechanism of priming–purgation–restoration. This integrated mechanism not only optimized the effectiveness of bowel preparation but also reduced inflammatory responses and promoted gastrointestinal motility, thereby synergistically enhancing the overall implementation of the ERAS pathway. Furthermore, by deeply integrating nutritional intervention with multidisciplinary ERAS management, this study highlights the collective value of collaboration among surgery, nursing, nutrition, and anesthesiology in improving perioperative experience and recovery quality. Based on these findings, the sequential protocol shows strong potential for generalizability and clinical application in day-case surgery and other short-stay surgical settings.

### 4.5 Limitations and future perspectives

This study has several limitations. First, as a single-center study with a relatively limited sample size, the generalizability and external validity of our findings require confirmation in multicenter, large-scale randomized controlled trials. Second, this research primarily evaluated the sequential olive oil–lactulose protocol from a clinical outcome perspective, without dynamic monitoring of gut microbiota composition, metabolic products (e.g., short-chain fatty acids), or inflammatory markers; therefore, mechanistic evidence remains insufficient. Future studies could incorporate serial stool sampling (e.g., baseline, preoperative after completion of the protocol, and postoperative day 1–7), 16S rRNA sequencing/metagenomics, targeted SCFA quantification, and perioperative inflammatory markers to validate the proposed microbiota-metabolite-recovery pathway.

Third, the bowel-preparation regimens differed in duration (single-day PEG vs. multi-day sequential protocol), and preoperative adherence was assessed mainly by patient self-report in a day-case setting; thus, adherence-related measurement bias and differential compliance cannot be completely ruled out. Standardized adherence monitoring (e.g., electronic diaries, supervised dosing when feasible, or verification of returned sachets/bottles) should be considered in future trials. Fourth, although both groups followed the same standardized low-residue/low-fiber diet instruction during the 3 days before surgery, habitual dietary patterns (e.g., predominantly vegetarian/high-fiber intake) were not formally recorded, and baseline fecal loading/retention was not routinely evaluated by plain abdominal radiography; therefore, we were unable to perform stratified analyses in patients with constipation or marked fecal loading, which may modify bowel-function outcomes (32). In addition, use of GLP-1 receptor agonists was not systematically recorded; given that GLP-1RA exposure may delay transit and has been associated with altered bowel preparation quality, future studies should collect GLP-1RA use and perform stratified analyses accordingly (8).

Finally, SIBO status and lactulose-related cramping were not assessed. We did not systematically screen for SIBO (e.g., breath testing) or record/grade lactulose-related colicky abdominal pain as a predefined adverse-event endpoint. Because lactulose efficacy and symptom profile depend on bacterial fermentation, future work should incorporate baseline symptom questionnaires, SIBO risk assessment (and testing when appropriate), and standardized recording of abdominal cramping/bloating to enable stratified analyses.

Future investigations could incorporate 16S rRNA gene sequencing, metagenomics, and metabolomics to elucidate the roles of olive oil and lactulose in modulating gut microecology and inflammatory responses. A feasible approach is serial stool sampling (e.g., baseline, preoperative after completion of the protocol, and postoperative day 1–7) with targeted quantification of fecal/serum SCFAs, combined with perioperative inflammatory markers and symptom scores. Correlating these biomarkers with clinical endpoints (time to first flatus/defecation, abdominal distension, nausea/vomiting, pain, and length of stay) would help validate the proposed lactulose–microbiota–recovery pathway. In addition, although this protocol was better tolerated than PEG, some patients reported difficulty accepting the direct oral intake of olive oil due to its taste. To address this, future work may explore emulsification techniques, capsule formulations, or combination with other nutritional supplements to develop more palatable preparations and improve compliance (33). Finally, with the advancement of digital health, smart follow-up platforms and mobile health applications could be leveraged to achieve more efficient and precise perioperative management in day-case surgery.

## 5 Conclusions

The sequential olive oil-lactulose protocol integrated within the ERAS pathway significantly improved bowel-preparation compliance and tolerance in patients undergoing day-case laparoscopic inguinal hernia repair, while reducing complications, accelerating gastrointestinal recovery, shortening hospital stay, alleviating pain, and enhancing patient satisfaction. Through a continuous “preoperative priming—preoperative evacuation—postoperative repair” mechanism, this approach demonstrated superior clinical value compared with the conventional PEG regimen and warrants further validation and broader application.

## Data Availability

The original contributions presented in the study are included in the article/Supplementary Material, further inquiries can be directed to the corresponding author.
